# A structure-based high-efficiency homogeneous antibody platform by endoglycosidase Sz provides insights into its transglycosylation mechanism

**DOI:** 10.1063/4.0001105

**Published:** 2025-10-27

**Authors:** Chun-Jung Chen

**Affiliations:** National Synchrotron Radiation Research Center, Hsinchu, N/A, Taiwan

## Abstract

Monoclonal antibodies (mAbs) have gradually dominated the drug markets for various diseases. Improvement of the therapeutic activities of mAbs has become a critical issue in the pharmaceutical industry. A novel Endo-b-*N*-acetylglucosaminidase, EndoSz, from *Streptococcus equi subsp. zooepidemicus Sz105* was discovered and applied to enhance the activities of mAbs. Our studies demonstrate that the mutant EndoSz- D234M possesses an excellent transglycosylation activity to generate diverse glycoconjugates on mAbs. We prove that EndoSz-D234M can be applied to various marketed therapeutic antibodies and those in development for antibody remodeling. The remodeled homogeneous antibodies (mAb-G2S2) produced by EndoSz-D234M increase the relative ADCC activities by 3–26 folds. We further report the high-resolution crystal structures of EndoSz- D234M in the *apo*-form and the complex form with a bound G2S2-oxazoline intermediate. A novel pH-jump method was utilized to obtain the complex structure with a high resolution. The detailed interactions of EndoSz-D234M and the carried G2S2-oxazoline are hence delineated. The oxazoline sits in a hole, named the oxa-hole, which stabilizes the G2S2-oxazoline in transit and catalyzes the further transglycosylation reaction while targeting Asn-GlcNAc (+1) of Fc. In the oxa-hole, the H-bonding network involved with oxazoline dominates the transglycosylation activity. A mobile loop 2 of EndoSz-D234M reshapes the binding grooves for the accommodation of G2S2- oxazoline upon binding, at which Trp154 forms a hydrogen bond with Man (–2). The long loop 4 followed by the helix 3 is capable of dominating the substrate selectivity of EndoSz-D234M. In addition, the stepwise transglycosylation behavior of EndoSz-D234M is elucidated. Based on the high-resolution structures of the *apo*- form and bound- form with G2S2-oxazoline as well as a systematic mutagenesis study of the relative transglycosylation activity, the transglycosylation mechanism of EndoSz-D234M is revealed.

